# Minimal pre-operative leg length discrepancy as a risk factor of post-operative leg length discrepancy after total hip arthroplasty: a retrospective study of patients with non-traumatic osteonecrosis of the femoral head

**DOI:** 10.1186/s12891-023-07086-2

**Published:** 2023-12-08

**Authors:** Hong Seok Kim, Han Jin Lee, Jeong Joon Yoo

**Affiliations:** 1 Department of Orthopedic Surgery, Seoul National University Hospital, Seoul, South Korea; 2 Department of Orthopedic Surgery, College of Medicine, Seoul National University, Seoul, South Korea; 3 Department of Orthopedic Surgery, Hanil General Hospital, Seoul, South Korea

**Keywords:** Pre-operative leg length discrepancy, Proximal femoral shape, Osteonecrosis of the femoral head, Unilateral total hip arthroplasty

## Abstract

**Background:**

Leg length discrepancy (LLD) is one of the troublesome complications of total hip arthroplasty (THA). Previously, several risk factors have been suggested, but they were subjected to their inherent limitations. By controlling confounding variables, we hypothesized that known risk factors be re-evaluated and novel ones be discovered. This study aimed to analyze the independent risk factors for LLD after primary THA in patients with non-traumatic osteonecrosis of the femoral head (ONFH).

**Methods:**

We retrospectively reviewed patients with non-traumatic ONFH who underwent unilateral THA between 2014 and 2021. All patients were operated by one senior surgeon using a single implant. Demographic data, surgical parameters, and radiological findings (pre-operative LLD, Dorr classification, and femoral neck resection) were analyzed to identify the risk factors of ≥ 5 mm post-operative LLD based on radiological measurement and to calculate odds ratios by logistic regression analysis. Post hoc power analysis demonstrated that the number of analyzed patients was sufficient with 80% power.

**Results:**

One hundred and eighty-six patients were analyzed, including 96 females, with a mean age of 58.8 years at the time of initial THA. The average post-operative LLD was 1.2 ± 2.9 mm in the control group and 9.7 ± 3.2 mm in the LLD group, respectively. The LLD group tended to have minimal pre-operative LLD than the control group (-3.2 ± 5.1 mm vs. -7.9 ± 5.8 mm *p* = 2.38 × 10^− 8^). No significant difference was found between the groups in age, gender, body mass index, femoral cortical index, and implant size.

**Conclusion:**

Mild pre-operative LLD is associated with an increased risk of post-operative LLD after primary THA in patients with ONFH. Thus, surgeons should recognize pre-operative LLD to achieve an optimal outcome and must inform patients about the risk of developing LLD.

**Supplementary Information:**

The online version contains supplementary material available at 10.1186/s12891-023-07086-2.

## Introduction

As the longevity of total hip arthroplasty (THA) implants has been continuously increasing, [1] so has the number of patients who underwent THA [2]. Leg length discrepancy (LLD) after THA has been not uncommonly reported. Perceived post-operative LLD-impaired patients’ function [3], limping [4], and patient dissatisfaction [5], leading to legal issues [6, 7].

To prevent this complication, several previous studies have elucidated the possible risk factors for post-operative LLD. Brumat et al. and Lim et al. suggested that the shape of the proximal femur and post-operative LLD were related [8, 9]. In revision THA, Kennedy et al. revealed that body mass index (BMI) was positively related to the risk of developing post-operative LLD [10]. Furthermore, Kishimoto et al. interestingly suggested that surgeon volume was associated with the risk of post-operative LLD [11]. However, these studies were limited to numerous pre-operative diagnoses, various types of cemented and cementless femoral stems, and several surgeons participating in the studies. Thus, it is hard to know how much these factors affect the outcome of surgery.

Osteonecrosis of the femoral head (ONFH) is a prevalent indication of THA [12]. Patients diagnosed with ONFH typically presented with a younger age and relatively mild deformity of the proximal femur, making this cohort more sensitive to LLD. Consequently, a thorough understanding of postoperative LLD and its associated risk factors in ONFH patients undergoing THA is essential for optimizing surgical outcomes and enhancing the overall quality of patient care.

We hypothesized that by controlling the confounding variables, previously reported possible risk factors might be re-evaluated and novel risk factors could be discovered. The aim of this study was to evaluate the risk factors of post-operative LLD (1) by controlling pre-operative diagnosis for THA, (2) by using the same THA implant, and (3) by performing the surgery with a single senior surgeon.

## Methods

### Study design

**Fig. 1:**
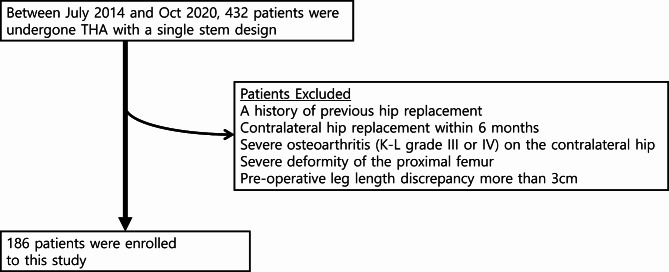
Flow-chart of enrolled patients

This was a retrospective observational study that followed STROBE guideline [13]. Upon Institutional Review Board approval, we conducted a study on a cohort of patients with uni-lateral non-traumatic osteonecrosis of the femoral head who had primary THA with a single stem design. We included patients who received primary THA with the studied implant in a tertiary referral hospital from July 2014 to Oct 2020. Patients were excluded if they had a history of previous hip replacement on the contralateral hip, had the contralateral hip replacement within 6 months, and had severe osteoarthritis on the contralateral hip joint, severe deformity of the proximal femur, or a pre-operative leg length discrepancy of more than 3 cm (Fig. 1). Among the analyzed patients, the patients whose post-operative LLD were equal to or more than 5 mm were grouped as an LLD group [8, 14, 15], while the others were classified as a control group.

### Operation

**Fig. 2:**
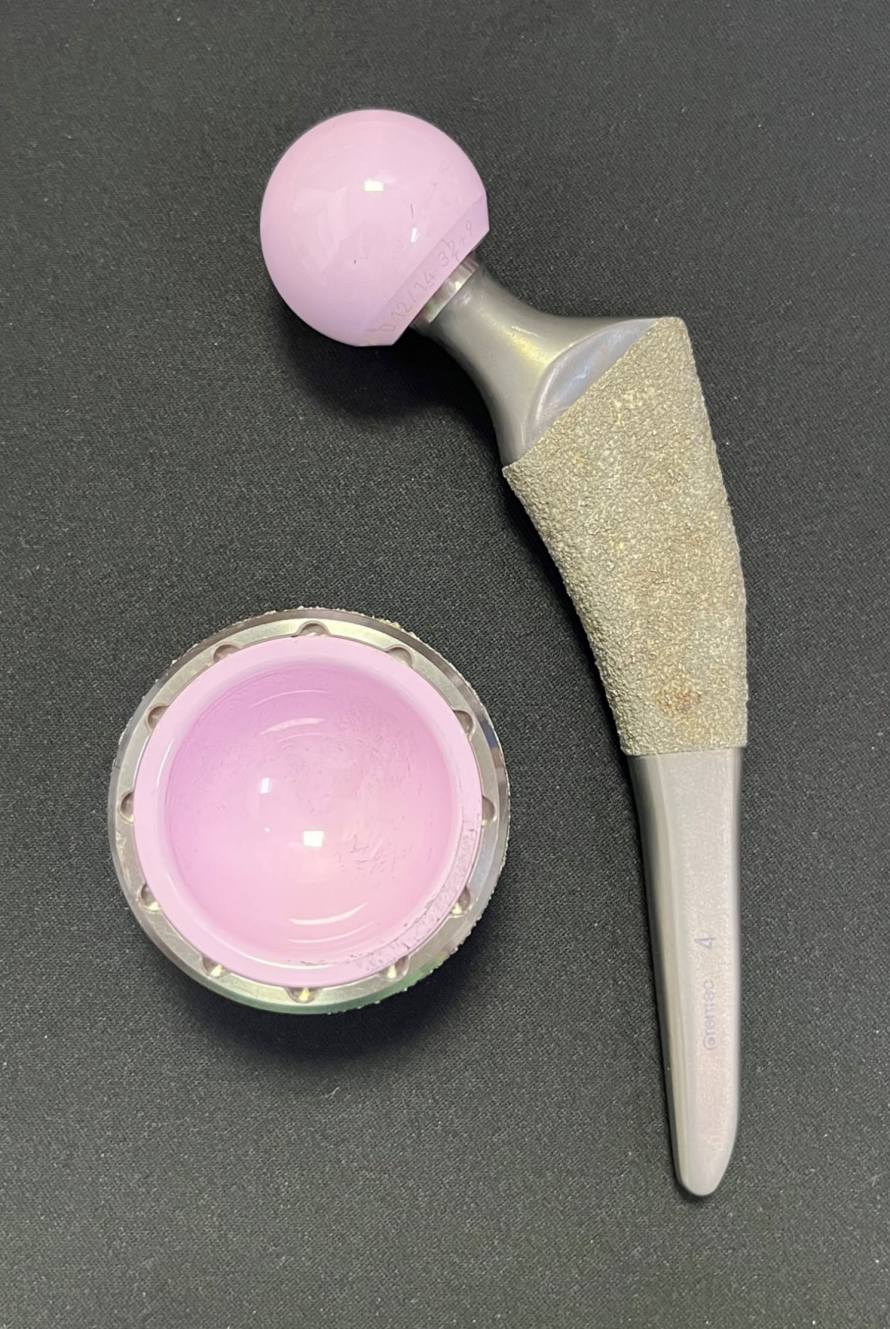
Bencox Mirabo cup with M femoral stem (Corentec, Cheonan, Korea) and Biolox Delta bearing (Ceramtec, Plochingen, Germany) were used in this study

All operations were performed by a high- volume hip arthroplasty surgeon (JJY) with more than 20 years of experience using the modified direct lateral approach [16]. One type of cementless THA implant and bearing was used: Bencox Mirabo acetabular cup with Bencox M stem (Corentec, Cheonan, Korea) and alumina-zirconia composite Biolox Delta (CeramTec, Plochingen, Germany) (Fig. 2). The stem is a type I stem that is widely used [17] and features a single wedged, proximally coated, and mid-short length [18].

Preoperative acetate onlay templating was performed in all patients. Templating was used to guide the femoral neck resection level, choice of implant size, and implant position. However, the final decision was based on the intraoperative features of the patients. To make more accurate decisions, intraoperative anteroposterior pelvic radiographs were also routinely taken via cross-table manner right after the insertion of implant trials.

Patients were instructed to walk using crutches with tolerable weight-bearing for post-operative 6 weeks.

### Clinical variables

The following data were obtained: age at index surgery, gender, pre-operative diagnosis, BMI, and pre-operative radiological leg length discrepancy.

### Radiologic evaluation and analysis

Hip anteroposterior view was taken while standing with 15° internally rotated legs when the beam was incident on the median line 2.5cm above the pubic symphysis [19].

To validate the accuracy of the PACS (INFINITT PACS Viewer, Seoul, South Korea) measuring method, we first draw a circle that perfectly fits the acetabular cup and measured the diameter. Then, the measurements were compared with the actual implant, yielding the ratio between the actual value and the PACS viewer measured value.

Two independent authors (HSK, HJL) who did not participate in the index surgery performed all measurements. The authors repeated the measurement 6 weeks later, and the mean difference was 0.6 ± 0.3 mm with intra-observer reliability of 0.945. Inter-observer reliability using the intra-class correlation coefficient was 0.843. The definition of ICC values was as follows: excellent reliability (> 0.90), good reliability (0.75–0.90), moderate reliability (0.50–0.75), and poor reliability (< 0.50) [20].

### Femoral cortical index

On an anteroposterior radiograph of the hip, the femoral cortical index (FCI) was defined as the ratio of cortical width minus canal width to the cortical width at a level of 100 mm below the apex of the lesser trochanter, as described in previous studies [21]. These measurements were repeated 6 weeks later to assess the intra-observer reliability. The mean difference in FCI was 0.04 ± 0.02 and the intra-observer reliability was 0.875. All patients were categorized on the basis of FCI with the cutoff values as follows: >0.6 as Dorr type A, between 0.6 and 0.5 as Dorr type B, and < 0.5 as Dorr type C [9].

### Leg length discrepancy

The leg length discrepancy was defined as the perpendicular distance between the lesser trochanters and a horizontal reference line connecting the inferior aspect of the acetabular teardrops on an anteroposterior radiograph of the pelvis at least 6 months after THA [22] (Supplementary Fig. 1). The measurements were repeated 6 weeks later to assess the intra-observer reliability in the corresponding technique. The mean difference in LLD was 0.5 ± 0.4 mm and the intra-observer reliability was 0.902.

### Femoral neck resection and cup center level

The femoral neck resection level was measured as the vertical distance from the tip of the lesser trochanter to the bone resection cut on the medial side of the femoral neck. The level of the cup center relative to the greater trochanter was defined as the vertical distance from the tip of the greater trochanter to the cup center.

### Statistical analyses

SPSS statistics for Windows (Version 25.0; IBM, Armonk, NY) was used for the statistical analyses. All continuous variables are reported as mean and standard deviation, and categorical variables are reported as numbers and percentages. A Student t-test or Mann-Whitney U test for continuous variables and the chi-square test or Fisher’s exact test for categorical variables were performed. Multiple logistic regression analysis was performed to identify the independent risk factors of post-operative LLD. Significance was defined as a *p*-value of less than 0.05.

## Results

In total, 186 patients were analyzed in this study. Pre-operative and post-operative 6-month radiographs of all patients were examined. An absolute LLD ≥ 5 mm after THA was observed in 45.2% of the patients (84 of 186 hips). The mean post-operative LLD was 5.1 ± 5.2 mm, and the mean FCI was 0.54 ± 0.06. There was no significant difference between the two groups with regard to age, gender, BMI, operated site, and the cause of non-traumatic osteonecrosis of the femoral head (Table 1). A post-hoc analysis was conducted and confirmed that the number of analyzed patients was larger than the minimum sample size with 80% power and an alpha of 0.05.

**Table 1:** Patient demographic of 186 hips

Parameter		Cases; LLD > = 5 mm	Controls; LLD < 5 mm	*p*-value
Number	n	84	102	
Post-operative LLD (mm)	Mean (SD)	9.7 (3.2)	1.2 (2.9)	< 0.001
Gender	n (%)			
Female		44 (52.4%)	46 (45.1%)	0.323
Male		40 (47.6%)	56 (54.9%)	
Age (years)	Mean (SD)	58.4 (12.4)	59.1 (13.1)	0.739
BMI (kg/m^2^)	Mean (SD)	24.2 (3.8)	24.6 (3.8)	0.534
Laterality	n (%)			
Right		46 (54.8%)	57 (55.9%)	0.878
Left		38 (45.2%)	45 (44.1%)	
Cause of Osteonecrosis	n (%)			
Alcohol abuse		22 (26.2%)	30 (29.4%)	0.544
Use of glucocorticoids		9 (10.7%)	12 (11.8%)	
Sickle cell anemia		0 (0%)	2 (2.0%)	
Systemic lupus erythematosus		0 (0%)	1 (1.0%)	
Radiation treatment		0 (0%)	1 (1.0%)	
Unidentified		53 (63.1%)	56 (54.9%)	
Dorr classification	n (%)			
Type A		17 (20.2%)	13 (12.7%)	0.292
Type B		46 (54.8%)	56 (54.9%)	
Type C		21 (25.0%)	33 (32.4%)	
Pre-operative LLD (mm)	Mean (SD)	-3.2 (5.1)	-7.9 (5.8)	< 0.001
Femoral neck resection (mm)	Mean (SD)	20.6 (4.6)	20.8 (5.9)	0.734
Acetabular cup size	Median [range]	52 [46, 62]	52 [46, 62]	0.052
Femoral stem size	Median [range]	4 [1, 10]	5 [1, 10]	0.087
Ceramic head size	n (%)			0.143
28 mm		42 (50.0%)	43 (42.2%)	
32 mm		36 (42.9%)	45 (44.1%)	
36 mm		6 (7.1%)	14 (13.7%)	
Neck length	n (%)			0.906
Short		1 (1.2%)	2 (2.0%)	
Medium		68 (81.0%)	81 (79.4%)	
Long		15(17.9%)	19 (18.6%)	
The amount of lengthening (mm)	Mean (SD)	12.9 (5.3)	9.2 (6.0)	< 0.001
Cup center respect to GT (mm)	Median [range]	0.0 [-8.9, 27.6]	-1.4 [-18.7, 10.3]	0.164

Abbreviations: LLD = leg length discrepancy, SD = standard deviation, GT = greater trochanter

**Fig. 3:**
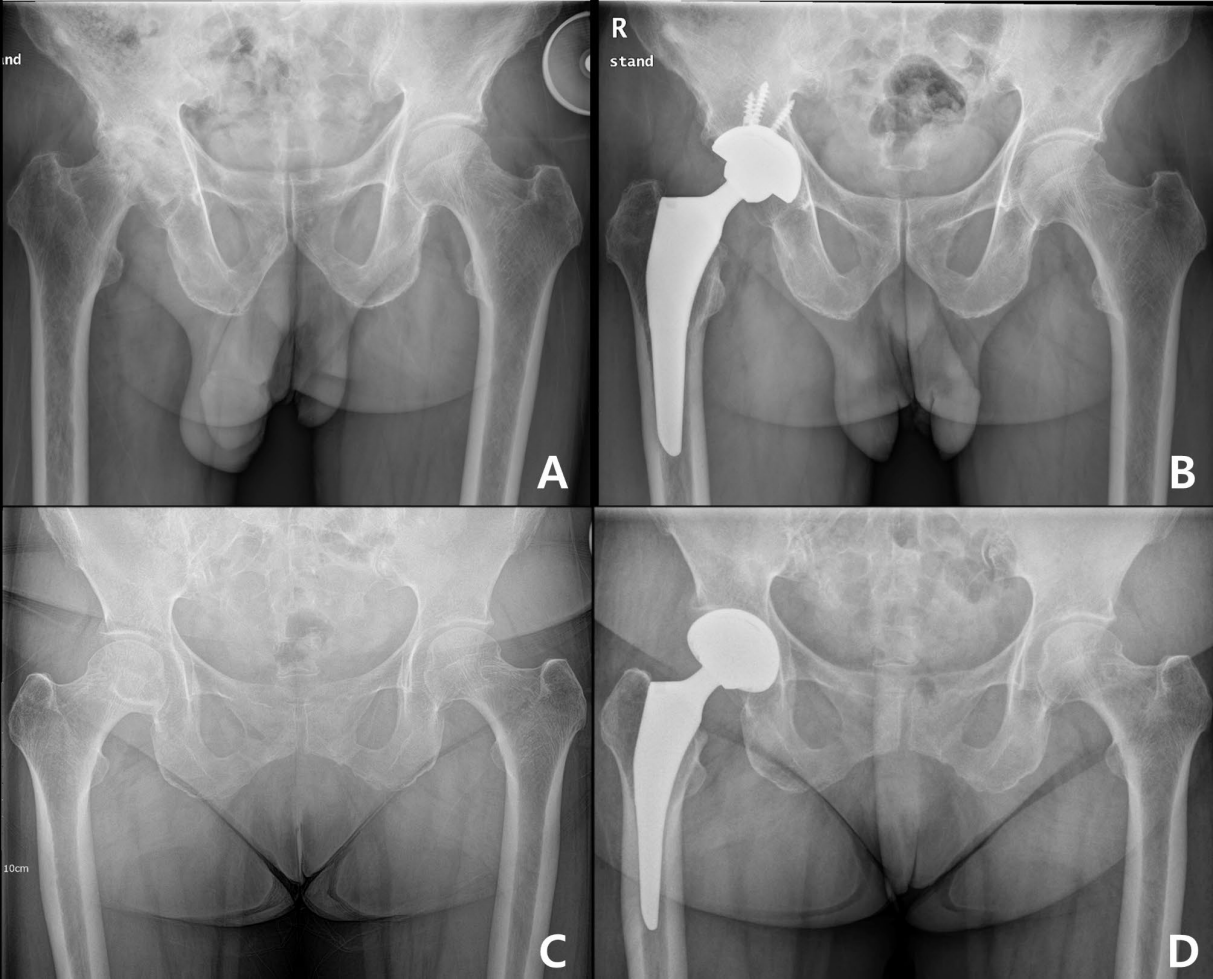
**(A)** Preoperative hip radiograph with substantial leg length discrepancy **(B) **Hip radiograph at postoperative 6 weeks with minimal leg length discrepancy **(C)** Simple hip radiograph with minimal preoperative leg length discrepancy **(D)** Postoperative 6 weeks radiograph with leg length discrepancy more than 5 mm

Minimal LLD before surgery was more likely to be associated with LLD ≥ 5 mm after surgery. The pre-operative LLD of the LLD group was − 3.2 ± 5.1 mm, whereas that of the control group was − 7.9 ± 5.8 mm (*p* < 0.001) (Fig. 3).

The shape of the proximal femur, which was represented by FCI, was not associated with the post-operative LLD (*p* = 0.292). In terms of implant size choice, no significant difference was found between the two cohorts in acetabular cup size (*p* = 0.052), femoral stem size (*p* = 0.087), head size (*p* = 0.143), and dependent neck length (*p* = 0.906). In addition, the amount of femoral neck resection and cup center level with respect to the greater trochanter showed little difference between the groups (*p* = 0.734 and *p* = 0.164, respectively) (Table 1).

Among eighty-eight patients with minimal LLD (less than 5 mm) preoperatively, the ratio of patients with LLD ≥ 5 mm after surgery was 61.4% (54 of 88). It was higher than that within the patients with preoperative substantial LLD, which was 30.6% (30 of 98) (*p* < 0.001).

Possible risk factors from the univariate analysis were included in the multiple logistic regression analysis. The analysis showed that pre-operative LLD (*p*-value < 0.01) was the only independent risk factor associated with post-operative LLD.

The average follow-up period was similar in both groups (2.5 ± 1.9 versus 2.5 ± 1.8 years; *p* = 0.873).

## Discussion

The present findings of this study indicated that the patient with preoperatively balanced leg length showed more imbalance after the surgery and the patient who suffered from substantial difference in limb length resulted in little difference in limb length (Fig. 3). Among other complications, LLD after THA was considered the most troublesome complication, which was directly related to patient satisfaction after surgery and was hard to resolve. Since the successful THA should have complication-free long-term survival and acceptable patient satisfaction, the surgeon should take account of pre-operative LLD.

The presented findings on the association between pre-operative and post-operative LLD have direct clinical applications for THA surgery. Minimal pre-operative LLD led to more post-operative LLD and should be considered as a risk factor for post-operative LLD in cementless type I stems. No significant difference was found between the groups in age, gender, body mass index, femoral cortical index, and implant size. Additional intraoperative procedures such as the usage of high offset stem, additional medullary preparation, or intraoperative radiograph would be needed.

Previously, a broad consensus was accepted that ≤ 10 mm of LLD on radiographs was clinically irrelevant [23, 24]. To date, the definitions of LLD varied based on radiological measurements: 5 mm [8, 14, 15, 25], 6 mm [26], 7 mm [27], 10 mm [28, 29], and 20 mm [30]. Sykes et al. [25] demonstrated that a large number of patients with discrepancies ≥ 5 mm had perceived the difference, and Renkawitz et al. [15] showed that gait kinematics might be induced due to discrepancies ≥ 5 mm. Fujita et al. showed that 7 mm may be a reasonable threshold for reducing residual discomfort [27]. In our study,a cut-off value of 5 mm was used to define LLD after surgery, and the proportion of post-operative LLD was high in patients with minimal pre-operative LLD. To the best of our knowledge, our study is the first to suggest the importance of pre-operative LLD.

Previous studies have suggested possible risk factors for post-operative LLD. In 2019, Mavčič et al. analyzed the effects of gender, age, operated side, surgical approach, body height, and BMI. The authors concluded that patients with smaller body dimensions (i.e. low BMI and small height) will be more likely to report subjective leg length inequality at a given objective LLD, regardless of gender or age [31]. Kishimoto et al. reported that surgeon volume might be an independent risk factor for leg length discrepancy. 79.6% of patients performed by high-volume surgeons had LLD of < 5 mm, while 40.0% of patients performed by low-volume surgeons achieved LLD of < 5 mm (*p* < 0.001). [11] However, previous studies included various stem designs, several surgeons performing the surgeries, and diverse pre-operative diagnoses. In this study, we designed the study to control these parameters: BMI, height, gender, and age did not influence leg length inequality after surgery. In addition, all index surgeries were performed by high-volume hip surgeons in our study.

Then, intra-operative factors, such as soft tissue laxity or hip flexion contracture, should be investigated. Contracture and adhesion around the hip joint due to the shortened limb could impede appropriate leg lengthening even with extensive soft tissue release. The previous literature has reported that patients with developmental dysplasia of the hip frequently present with moderate flexion contracture of the hip joint, necessitating additional soft tissue release during total hip arthroplasty [32, 33]. In our study, the amount of lengthening in patients with a significant preoperative LLD was shorter than that in the cohort with minimal preoperative LLD. While patients with ONFH tended to exhibit fewer developmental deformities, shortened limb may still lead to soft tissue contracture over time. In contrast, patients with minimal LLD may experience less joint contracture, which prompt surgeons to select larger implants or longer neck components for improved stability. Further studies analyzing the discrepancy between the pre-operative templating and the actual implant used should consider these concerns.

Among other risk factors, the morphology of the proximal femur has been considered important and discussed several times. Brumat et al. [8] concluded that a higher femoral canal flare index increased LLD after surgery when single-wedge femoral stems with cementless metaphyseal fixation were used. With cementless diaphyseal fixation or cemented fixation, however, the canal flare index had no impact on LLD in femoral stems. Lim et al. [9] have also shown that the proximal femoral canal morphology affected LLD. This study utilized more than three different stems, and the index surgeries were performed by three surgeons. In a recent systematic review by Mavčič et al. [34], the authors showed that Dorr type A (FCI > 0.6) increases the risk of leg length inequality after THA, especially when metaphyseal fixation stems were used. In the current study, where the same femoral stem was used and only one surgeon performed the operation, the Dorr type was not associated with post-operative LLD. This is comparable to the recent study of Kishimoto et al. [11] in which the Dorr type showed no association with post-operative LLD. We assumed that the differences in the study methods, surgical approach, and intraoperative medullary preparation might have affected the results.

Subjectively perceived LLD is important but was not analyzed in this study. Some patients would have complained with LLD less than 5 mm, while others would persevere the LLD more than 10 mm without any discomfort. Mavcic et al. [31] previously reported that patients with smaller body dimensions would more likely report subjective leg length inequality even within a given inequality. As so, our definition of LLD (≥ 5 mm) did not always match the patient’s dissatisfaction. However, generally perceived LLD was highly related to and based on objective LLD, which was studied here.

Preoperative templating and intraoperative radiographs would aid in decreasing the leg length discrepancy. We routinely performed preoperative conventional templating with digital PACS. Although the predetermined size of the stem may provide guidance in selecting the actual stem, the decision should be made comprehensively based on the clues in the intraoperative field. In 2018, Strøm and Reikerås reported that templating may be helpful but cannot be regarded as a blueprint for the operative choice [35]. Intraoperative fluoroscopy was also utilized, but care should be taken since the radiograph might be distorted depending on the patient’s posture, leg position, and magnification. Ezzet and McCauley analyzed the use of an intraoperative AP pelvic X-ray after trial component implantation, and found that the operative plan was altered in 50% of the surgeries, with changes being made to either the femoral alignment, leg length, cup medialization, or cup abduction [36]. Even with various perioperative measurements, absolute leg length equality is hard to achieve.

Limitations of this study were present. Firstly, only one disease entity was studied. The results might be different in THA for patients with trauma, infection sequelae, or dysplastic hip. A study with a larger cohort was warranted. Second, the studied stem might not be used in cases where the femoral cortical index is either very high or low. This would lead to selection bias. However, the range of FCI was from 0.35 to 0.67, and a substantial proportion of patients with high and low FCI was included. Lastly, the measurement was performed in plain radiographs. A slight change in patient position or the angle of beam insertion might influence the measured values. CT exam would provide an accurate assessment of limb length, but the cost and radiation hazards were concerns.

## Conclusion

This is the first study to show that minimal pre-operative LLD is associated with LLD after THA. The surgeon should implement additional intraoperative procedures such as the usage of a high offset stem, additional medullary preparation, or intraoperative radiograph to avoid post-operative LLD. The Dorr type did not affect post-operative LLD.

### Electronic supplementary material

Below is the link to the electronic supplementary material.

Supplementary Material 1

## Data Availability

The dataset supporting the conclusions of this article is included within the article.
