# Supplementary figures and images for: Minimal pre-operative leg length discrepancy as a risk factor of post-operative leg length discrepancy after total hip arthroplasty: a retrospective study of patients with non-traumatic osteonecrosis of the femoral head
Source: BMC Musculoskelet Disord. 2023 Dec 8;24:954. doi: 10.1186/s12891-023-07086-2 (PMC10704764; doi:10.1186/s12891-023-07086-2)

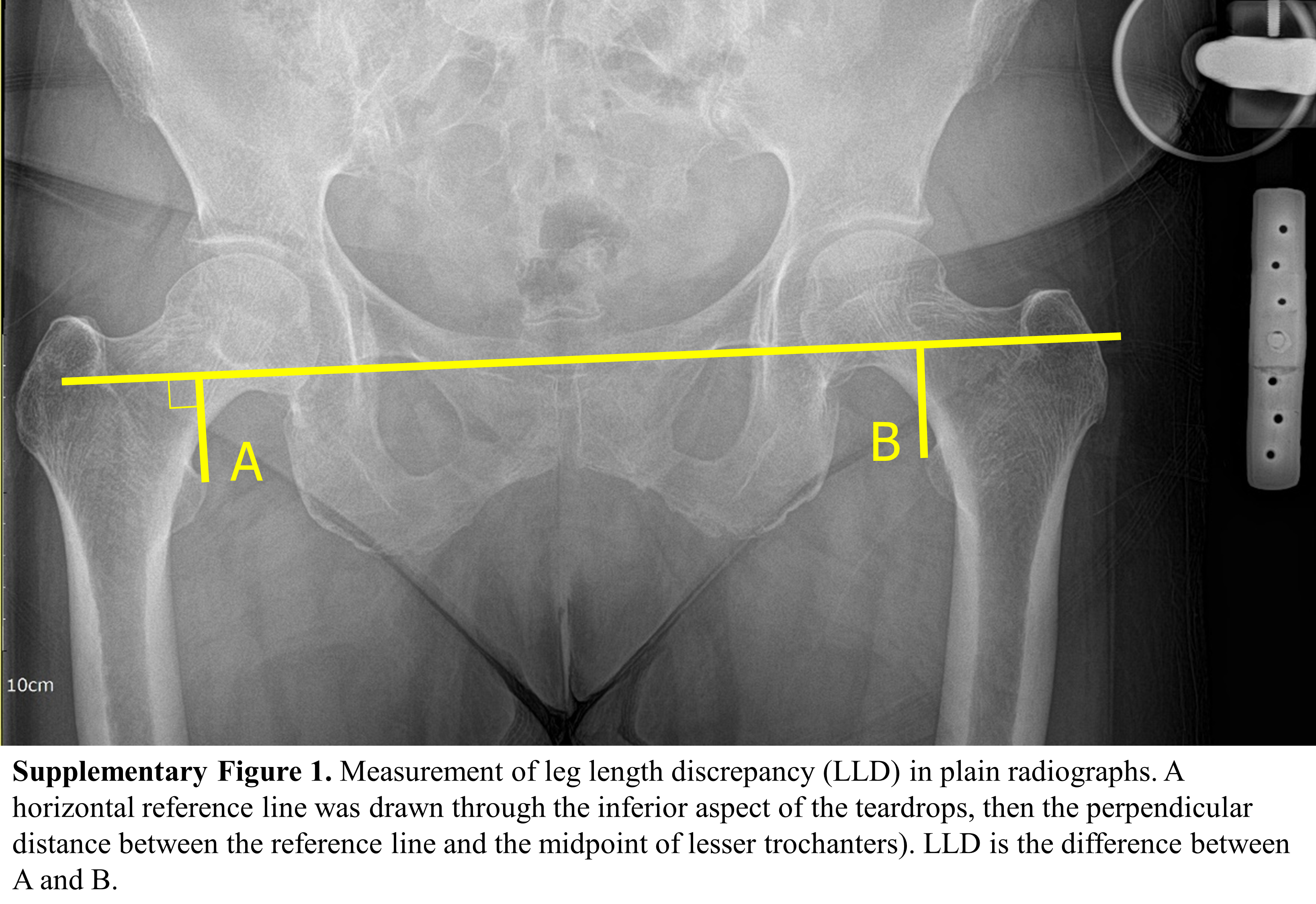

Supplement: Supplementary file 1 — Supplementary Material 1 [file 12891_2023_7086_MOESM1_ESM.tif]
